# Supplementary material for: A comprehensive map of human glucokinase variant activity
Source: Genome Biol. 2023 Apr 26;24:97. doi: 10.1186/s13059-023-02935-8 (PMC10131484; doi:10.1186/s13059-023-02935-8)

Fig. S1

GFP/GCK

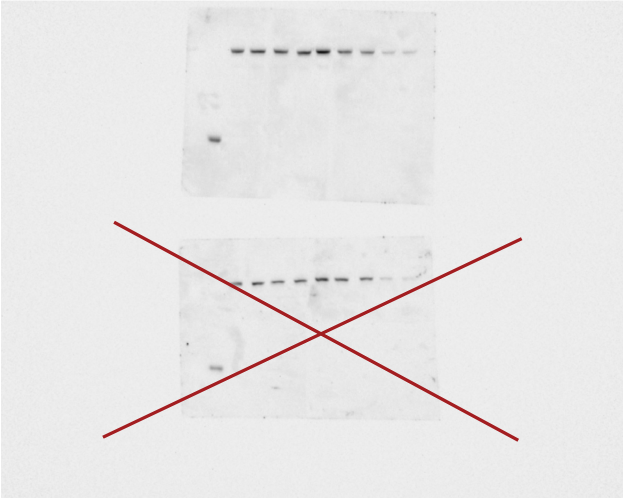

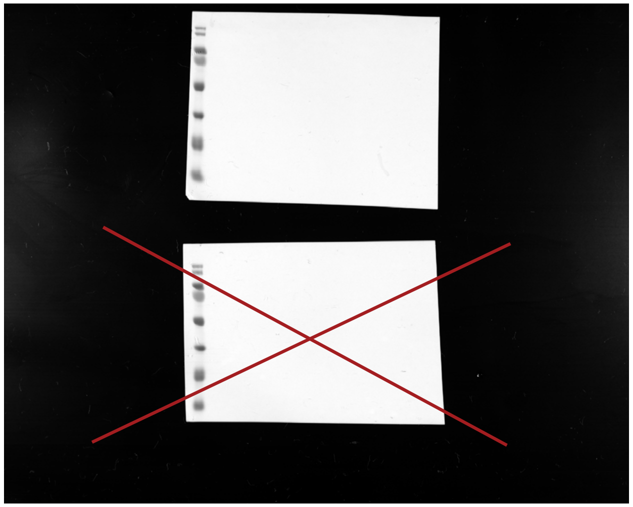

marker

merge

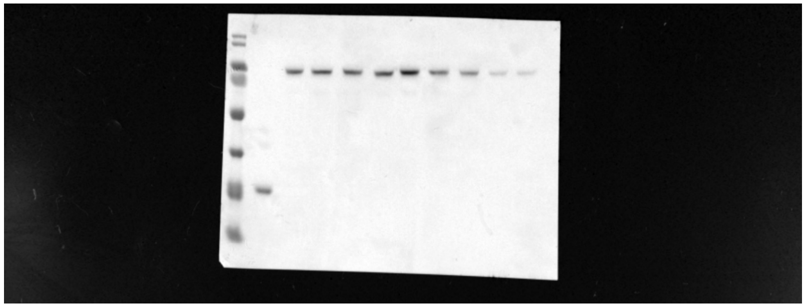

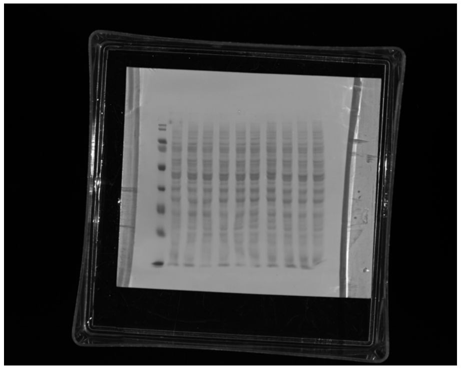

Ponceau S

Fig. S2

GFP/GCK

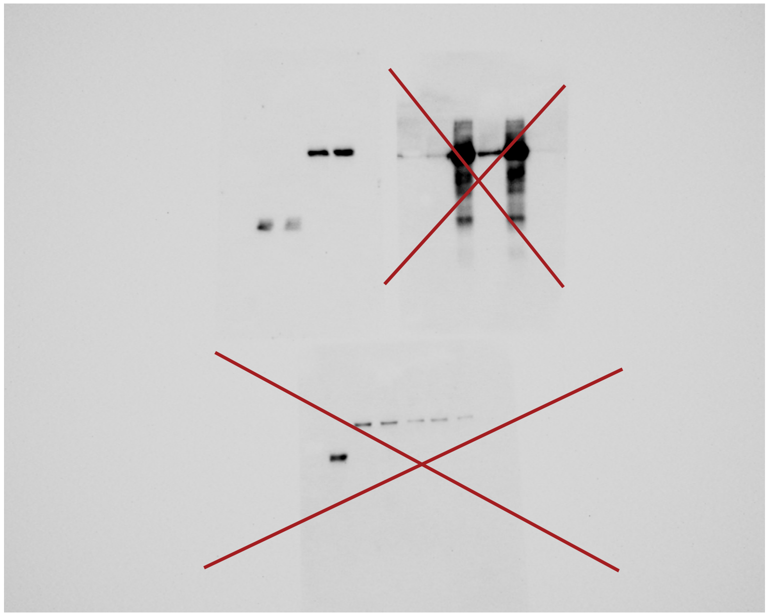

marker

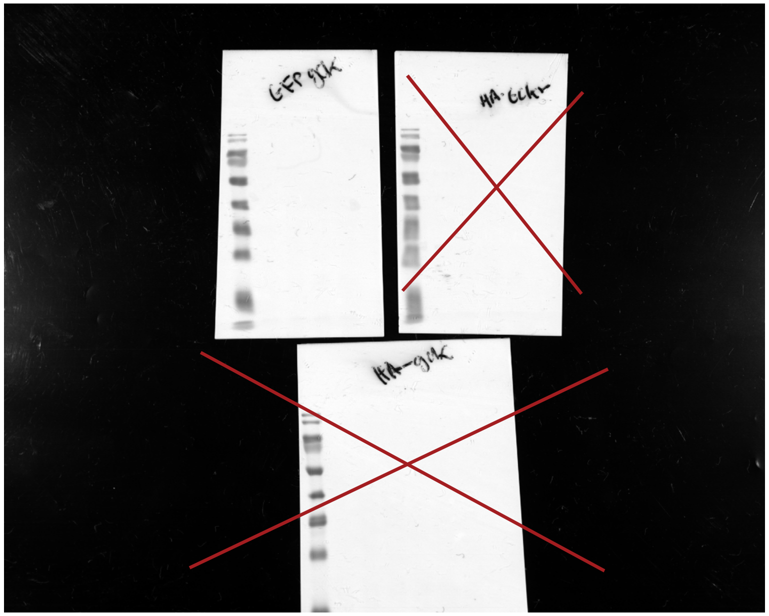

merge

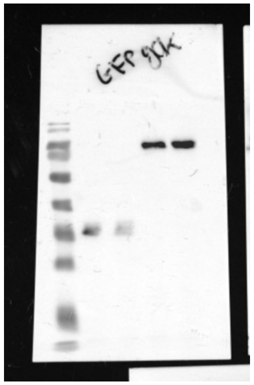

Ponceau S

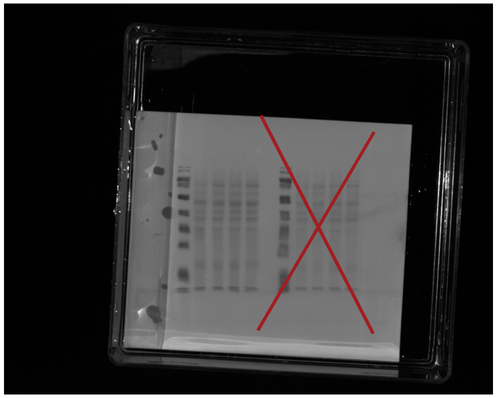

Fig. S2

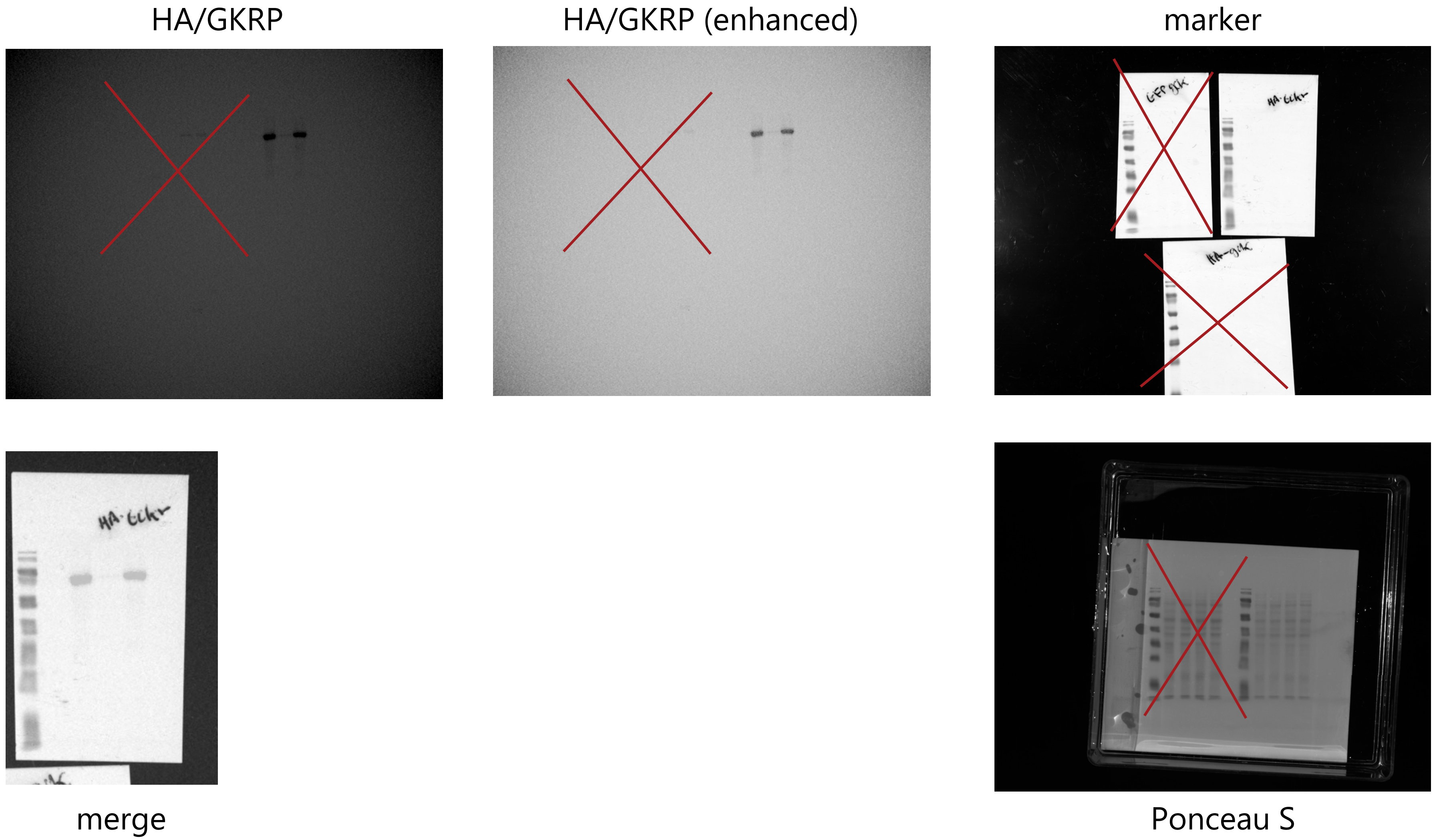

Supplement: Supplementary file 3 — Additional file 3. [file 13059_2023_2935_MOESM3_ESM.pdf]
