# Supplementary material for: A comprehensive map of human glucokinase variant activity
Source: Genome Biol. 2023 Apr 26;24:97. doi: 10.1186/s13059-023-02935-8 (PMC10131484; doi:10.1186/s13059-023-02935-8)
Supplement: Supplementary file 4 — Additional file 4. Review history. [file 13059_2023_2935_MOESM4_ESM.docx]

Review History

**First round of review**

**Reviewer 1**

**Were you able to assess all statistics in the manuscript, including the appropriateness of statistical tests used?** Yes, and I have assessed statistics in my report.

**Comments to author:**

Gersing, Roth, Lindorff-Larsen, Hartmann-Petersen and colleagues present the results of a mutational scan of human glucokinase (GCK) using a sequencing-based readout of a yeast growth assay. The authors generated a conditional yeast strain that required GCK activity for growth on glucose (but not galactose). They introduced human GCK variants on plasmids and demonstrated that under standard yeast growth conditions (0.2% glucose), growth rates for individual GCK variants correlated with kinetic measurements (kcat/Km). The high throughput sequencing results exhibited a somewhat surprising level of variation - for example, there appears to be some overlap between the activity scores of stop codons and synonymous variants. Despite the contributions of measurement noise, the high throughput activity measures correlate with kinetic measures showing that they capture trends well when averaged over multiple mutations. The authors discuss some factors that contribute to measurement noise including that variants can contain multiple mutations and the readout misses most linkage/epistasis. The authors harness the information in the mutational scan to investigate GCK mechanism. In my view, this is where this manuscript shines the brightest. The mutational data seems strong in identifying sensitive positions and larger scale trends. The authors use Rosetta computational modeling of mutations in two structural poses of GCK - super-open and closed. Through these analyses, the authors provide strong evidence in support of mechanistic models. The structural conclusions are convincing and interesting, including how mutations that disrupt the super-open conformation can lead to hyperactive GCK activity.

The manuscript is exceptionally well written, and it was a pleasure to read. The authors discuss shortcomings in their approach with an open-ness that is refreshing and useful. The mechanistic conclusions are interesting and well supported by the experimental results. From my perspective, the utility of this work in aiding the interpretation of human genetics is disappointing, and overly focused on in the manuscript. While I can see some benefit from the reported measures, the internal variation would frustrate me if I was trying to figure out a mutation in my genome or someone that I cared about. In my view, however, the experimental approach and the structural conclusions are fabulous and make this work highly appropriate for publication in Genome Biology. I have some minor comments and suggestions that are listed below.

1. Please clearly state if repeats of the mutational scan were performed. And if not, why?

2. Please say more about sources of variation in internal controls - including stop codons and synonymous mutations. It is disturbing that these classes of mutations appear to overlap. Please describe how the estimates of error were calculated without referring to previous work and also provide an analysis of how these errors predict the observed variation in synonyms and stop codons? Presumably, the error estimates account for sequencing depth. If there is additional error beyond sequencing depth, the authors could tie this nicely into their existing discussion of caveats such as secondary mutations.

3. Line 96 - how is GCK-MODY actionable? Please explain this in the intro.

4. The trend between conservation and experimental tolerance to mutation has been widely observed for other proteins. There is a nice review that covers the fundamental mechanisms that can contribute to this including the difference in scales of natural evolution compared to lab studies (Prot Sci 2016 Boucher et al PMC4918414). These points seem relevant to the current manuscript.

5. For future efforts, I would be excited to see analyses under different glucose concentrations, with the inhibitor protein, and potentially with different expression levels of GCK. However, I emplore the authors to first make the effort to improve the experimental system - add a random ~18 base barcode to the plasmid, use pac-bio CCS reads to associate the barcode with the entire ORF. Then use short read of the barcodes to quantify variant frequency in samples. It will make a world of difference in signal to noise, enable efficient readouts (including experimental repeats under multiple conditions). You are in the awesome position of being able to help people understand potential genetic disorders - precision in your measurements matters dearly.

**Reviewer 2**

**Were you able to assess all statistics in the manuscript, including the appropriateness of statistical tests used?** There are no statistics in the manuscript.

**Comments to author:**

This is a report of the use of a yeast-based synthetic biology model to devise a map of the functional effect of SNVs (missense and nonsense) on the function of GCK, the gene most often mutated in MODY. It is based on how well GCL variants can complement a KO of all three yeast hexokinases. This work represents an important application of large-scale functional annotation to a clinical problem. Although, in my opinion, the results do not quite make it to clinical utility, the concept is novel and interesting. The paper can be improved in a number of ways.

1. Lines 183-4: It is stated that "We used the standard deviation of synonymous variants (0.4) to set the thresholds". This statement makes no sense and does not appear to be what was actually done. The logical place to put the lowest limit of normal function would be at the delimitation between nonsense and synonymous variants which, in Fig. 2C looks like it is at 0.6, which is where the limit was actually placed later in the paper. The synonymous SD does seem to have been used for setting the threshold for the hyperactive variants at 1.4. This threshold is arbitrary and it does not make sense, as it includes a non-negligible portion of the synonymous variants. I believe that this problem explains the 14% of all variants being GOF, something implausible, especially considering the rarity of HH due to such GCK variants.

2. Line 225. How was the score imputed? The concept is not explained.

3. Line 234. Why so few benign variants? I presume that fasting blood glucose and GCK sequence was available from the 7k individuals (general population sample and the child cohort) used. If so, it is hard to believe that only three individuals with normal fasting glucose were found to have coding variants. If not, what was done with these cohorts and why are they even mentioned?

4. The evolutionary conservation analysis gives the impression of re-inventing the wheel, given the existence of multiple well established pathogenicity algorithms most of which rely extensively or exclusively on evolutionary conservation.

5. Lines 345-355 in the Discussion really belong under Methods. They give a perfectly good answer to the concern about multiple mutants on the same clone, which I would not consider a substantial weakness of the study. The excess of variant reads over the total number of tile reads can be used to calculate the frequency and redundancy of such multiple mutants. This text reads like an afterthought, in response to a previous review, and feels out of place in the Discussion.

6. Please annotate the horizontal series of points within the same concentration in each panel of Fig. 1. What do they represent?

7. Lines 212-213 The term "population" is used incorrectly. It should be "a population sample of Danish children (n=1138 . . ."

**Authors Response**

**Point-by-point responses to the reviewers’ comments:**

Reviewer #1: Gersing, Roth, Lindorff-Larsen, Hartmann-Petersen and colleagues present the results of a mutational scan of human glucokinase (GCK) using a sequencing-based readout of a yeast growth assay. The authors generated a conditional yeast strain that required GCK activity for growth on glucose (but not galactose). They introduced human GCK variants on plasmids and demonstrated that under standard yeast growth conditions (0.2% glucose), growth rates for individual GCK variants correlated with kinetic measurements (kcat/Km). The high throughput sequencing results exhibited a somewhat surprising level of variation - for example, there appears to be some overlap between the activity scores of stop codons and synonymous variants. Despite the contributions of measurement noise, the high throughput activity measures correlate with kinetic measures showing that they capture trends well when averaged over multiple mutations. The authors discuss some factors that contribute to measurement noise including that variants can contain multiple mutations and the readout misses most linkage/epistasis. The authors harness the information in the mutational scan to investigate GCK mechanism. In my view, this is where this manuscript shines the brightest. The mutational data seems strong in identifying sensitive positions and larger scale trends. The authors use Rosetta computational modeling of mutations in two structural poses of GCK - super-open and closed. Through these analyses, the authors provide strong evidence in support of mechanistic models. The structural conclusions are convincing and interesting, including how mutations that disrupt the super-open conformation can lead to hyperactive GCK activity.

The manuscript is exceptionally well written, and it was a pleasure to read. The authors discuss shortcomings in their approach with an open-ness that is refreshing and useful. The mechanistic conclusions are interesting and well supported by the experimental results. From my perspective, the utility of this work in aiding the interpretation of human genetics is disappointing, and overly focused on in the manuscript. While I can see some benefit from the reported measures, the internal variation would frustrate me if I was trying to figure out a mutation in my genome or someone that I cared about. In my view, however, the experimental approach and the structural conclusions are fabulous and make this work highly appropriate for publication in Genome Biology. I have some minor comments and suggestions that are listed below.

Our response:

We thank Dan for his time and thorough attention to our manuscript! To address both reviewers’ concerns regarding the clinical utility of the data, we have attempted to moderate the clinical focus throughout the manuscript (lines 32-34, 44-46, 103-104, 117-118, 405-408).

1. Please clearly state if repeats of the mutational scan were performed. And if not, why?

Our response:

We regret that the number of repeats was unclear, and have now clarified, both in the results (line 164) and methods (lines 543-544) sections, that two repeats of the selection were performed.

2. Please say more about sources of variation in internal controls - including stop codons and synonymous mutations. It is disturbing that these classes of mutations appear to overlap. Please describe how the estimates of error were calculated without referring to previous work and also provide an analysis of how these errors predict the observed variation in synonyms and stop codons? Presumably, the error estimates account for sequencing depth. If there is additional error beyond sequencing depth, the authors could tie this nicely into their existing discussion of caveats such as secondary mutations.

Our response:

This is a good point. We have now included a description of the error estimation (lines 589-599). In our discussion of caveats, we have included a discussion of the variation in nonsense and synonymous variants (lines 346-358).

3. Line 96 - how is GCK-MODY actionable? Please explain this in the intro.

Our response:

We apologize for this vague formulation and now describe how GCK-MODY is actionable in the beginning of the introduction (lines 56-61) and in the sentence at lines 100-103.

4. The trend between conservation and experimental tolerance to mutation has been widely observed for other proteins. There is a nice review that covers the fundamental mechanisms that can contribute to this including the difference in scales of natural evolution compared to lab studies (Prot Sci 2016 Boucher et al PMC4918414). These points seem relevant to the current manuscript.

Our response:

We agree that this trend is well-established, and have clarified this (lines 252-253). In addition, the review (ref. 69 in the revised manuscript) has some excellent points regarding the fitness effects of mutations that we have incorporated in the section on the analysis of evolutionary conservation (lines 275-278).

5. For future efforts, I would be excited to see analyses under different glucose concentrations, with the inhibitor protein, and potentially with different expression levels of GCK. However, I emplore the authors to first make the effort to improve the experimental system - add a random ~18 base barcode to the plasmid, use pac-bio CCS reads to associate the barcode with the entire ORF. Then use short read of the barcodes to quantify variant frequency in samples. It will make a world of difference in signal to noise, enable efficient readouts (including experimental repeats under multiple conditions). You are in the awesome position of being able to help people understand potential genetic disorders - precision in your measurements matters dearly. Our response:

We thank the reviewer for the kind offer. We completely agree that there are many theoretically appealing features of the ‘BarSeq’ approach, in which the full-length sequence of expression constructs is associated with flanking barcode sequences (e.g., using PacBio sequences). However, we also note that the alternative TileSeq approach used here can yield equivalent results, e.g. where both were performed for UBE2I (Weile et al., 2017). We also note that the widely acclaimed map for BRCA1 using saturation genome editing (Findlay et al., 2018), which is in active use at some of the largest clinical genetic diagnostic providers (e.g., Invitae and Ambry Genetics), used a method akin to TileSeq in the sense that full-length sequencing was not employed. Moreover, other publications have provided evidence of clinical utility for maps generated using the TileSeq strategy followed here (Floyd et al., 2023; Sun et al., 2020; Weile et al., 2021). That said, we continue to be excited to learn more about BarSeq, especially given that it has likely reached the tipping point of being the more economical approach.

Reviewer #2: This is a report of the use of a yeast-based synthetic biology model to devise a map of the functional effect of SNVs (missense and nonsense) on the function of GCK, the gene most often mutated in MODY. It is based on how well GCL variants can complement a KO of all three yeast hexokinases. This work represents an important application of large-scale functional annotation to a clinical problem. Although, in my opinion, the results do not quite make it to clinical utility, the concept is novel and interesting. The paper can be improved in a number of ways.

Our response:

Due to the concern regarding the clinical utility, we have moderated the clinical focus (lines 32-34, 44-46, 103-104, 117-118, 405-408), as also noted above for Reviewer 1.

1. Lines 183-4: It is stated that "We used the standard deviation of synonymous variants (0.4) to set the thresholds". This statement makes no sense and does not appear to be what was actually done. The logical place to put the lowest limit of normal function would be at the delimitation between nonsense and synonymous variants which, in Fig. 2C looks like it is at 0.6, which is where the limit was actually placed later in the paper. The synonymous SD does seem to have been used for setting the threshold for the hyperactive variants at 1.4. This threshold is arbitrary and it does not make sense, as it includes a non-negligible portion of the synonymous variants. I believe that this problem explains the 14% of all variants being GOF, something implausible, especially considering the rarity of HH due to such GCK variants.

Our response:

We agree that these thresholds, which are used in the beginning of the paper, were somewhat arbitrary and unnecessary, and have therefore deleted this section (line 177).

2. Line 225. How was the score imputed? The concept is not explained.

Our response:

We have clarified that the score was imputed using the Human Protein Variant Effect Map Imputation Toolkit (Wu et al., 2019) (lines 214-217), as noted previously in the manuscript (lines 172-174).

3. Line 234. Why so few benign variants? I presume that fasting blood glucose and GCK sequence was available from the 7k individuals (general population sample and the child cohort) used. If so, it is hard to believe that only three individuals with normal fasting glucose were found to have coding variants. If not, what was done with these cohorts and why are they even mentioned?

Our response:

These cohorts were only used to examine the correlation between fasting glucose and activity scores, and we did not classify the variants detected in the cohorts as either benign or pathogenic. The three benign variants used for ROC analyses were obtained from literature, and are the only known benign GCK variants. We have attempted to further clarify this (line(s) 211, 222-225).

4. The evolutionary conservation analysis gives the impression of re-inventing the wheel, given the existence of multiple well established pathogenicity algorithms most of which rely extensively or exclusively on evolutionary conservation.

Our response:

We apologize for giving this impression, which was not intended, and have clarified that evolutionary conservation analysis is widely used to assess the effect of protein variants (lines 252-253). Indeed, we and others have previously used GEMME for this kind of analysis, and a recent assessment of variant effect prediction tools against MAVE data shows it to be the second-best performing model out of the >30 models tested (https://www.proteingym.org/substitutions).

5. Lines 345-355 in the Discussion really belong under Methods. They give a perfectly good answer to the concern about multiple mutants on the same clone, which I would not consider a substantial weakness of the study. The excess of variant reads over the total number of tile reads can be used to calculate the frequency and redundancy of such multiple mutants. This text reads like an afterthought, in response to a previous review, and feels out of place in the Discussion.

Our response:

We have deleted a part of the extensive explanation of this limitation (lines 345-346) but prefer to retain the remainder of this section since it is linked to point 2 of reviewer #1 and our response to this issue.

6. Please annotate the horizontal series of points within the same concentration in each panel of Fig. 1. What do they represent?

Our response:

We apologize for missing annotations and have adapted the figure to clarify that the horizontal series of points are serial dilutions of yeast cells.

7. Lines 212-213 The term "population" is used incorrectly. It should be "a population sample of Danish children (n=1138 . . ."

Our response:

We have corrected the mistake (lines 203-204).

Findlay, G.M., Daza, R.M., Martin, B., Zhang, M.D., Leith, A.P., Gasperini, M., Janizek, J.D., Huang, X., Starita, L.M., and Shendure, J. (2018). Accurate classification of BRCA1 variants with saturation genome editing. Nature *562*, 217–222. https://doi.org/10.1038/S41586-018-0461-Z.

Floyd, B.J., Weile, J., Kannankeril, P.J., Glazer, A.M., Reuter, C.M., MacRae, C.A., Ashley, E.A., Roden, D.M., Roth, F.P., and Parikh, V.N. (2023). Proactive Variant Effect Mapping Aids Diagnosis in Pediatric Cardiac Arrest. Circ. Genomic Precis. Med. https://doi.org/10.1161/CIRCGEN.122.003792.

Sun, S., Weile, J., Verby, M., Wu, Y., Wang, Y., Cote, A.G., Fotiadou, I., Kitaygorodsky, J., Vidal, M., Rine, J., et al. (2020). A proactive genotype-to-patient-phenotype map for cystathionine beta-synthase. Genome Med. *12*, 1–18. https://doi.org/10.1186/S13073-020-0711-1/FIGURES/5.

Weile, J., Sun, S., Cote, A.G., Knapp, J., Verby, M., Mellor, J.C., Wu, Y., Pons, C., Wong, C., Lieshout, N. van, et al. (2017). A framework for exhaustively mapping functional missense variants. Mol. Syst. Biol. *13*, 957. https://doi.org/10.15252/MSB.20177908.

Weile, J., Kishore, N., Sun, S., Maaieh, R., Verby, M., Li, R., Fotiadou, I., Kitaygorodsky, J., Wu, Y., Holenstein, A., et al. (2021). Shifting landscapes of human MTHFR missense-variant effects. Am. J. Hum. Genet. *108*, 1283–1300. https://doi.org/10.1016/J.AJHG.2021.05.009.

Wu, Y., Weile, J., Cote, A.G., Sun, S., Knapp, J., Verby, M., and Roth, F.P. (2019). A web application and service for imputing and visualizing missense variant effect maps. Bioinformatics *35*, 3191–3193. https://doi.org/10.1093/BIOINFORMATICS/BTZ012.

**Second round of review**

**Reviewer 1:** The authors have addressed all of my concerns. I encourage publication.

**Reviewer 2:** This revised version adequately recognizes the shortcomings of the study, especially in terms of clinical utility.

I still think that the paper would read better if the text addressing the problem of >1 variants per clone is moved to Methods, but I leave this to the discretion of the authors and editors. I have no further comments on this submission.
